# Comparison of stereotactic radiotherapy and protons for uveal melanoma patients

**DOI:** 10.1016/j.phro.2024.100605

**Published:** 2024-06-26

**Authors:** Emmanuelle Fleury, Jean-Philippe Pignol, Emine Kiliç, Maaike Milder, Caroline van Rij, Nicole Naus, Serdar Yavuzyigitoglu, Wilhelm den Toom, Andras Zolnay, Kees Spruijt, Marco van Vulpen, Petra Trnková, Mischa Hoogeman

**Affiliations:** a Erasmus Medical Center Cancer Institute, University Medical Center, Department of Radiotherapy, Rotterdam, The Netherlands; b HollandPTC, Delft, The Netherlands; c College of Medicine, Al Faisal University, Riyadh, Saudi Arabia; d Erasmus Medical Center, Department of Ophthalmology, Rotterdam, The Netherlands; e Erasmus Medical Center, Department of Clinical Genetics, Rotterdam, The Netherlands; f Medical University of Vienna, Department of Radiation Oncology, Vienna, Austria

**Keywords:** Stereotactic radiotherapy, Proton therapy, Uveal melanoma, Imaging-based planning

## Abstract

**Background and purpose:**

Uveal melanoma (UM) is the most common primary ocular malignancy. We compared fractionated stereotactic radiotherapy (SRT) with proton therapy, including toxicity risks for UM patients.

**Materials and methods:**

For a total of 66 UM patients from a single center, SRT dose distributions were compared to protons using the same planning CT. Fourteen dose-volume parameters were compared in 2-Gy equivalent dose per fraction (EQD2). Four toxicity profiles were evaluated: maculopathy, optic-neuropathy, visual acuity impairment (Profile I); neovascular glaucoma (Profile II); radiation-induced retinopathy (Profile III); and dry-eye syndrome (Profile IV). For Profile III, retina Mercator maps were generated to visualize the geographical location of dose differences.

**Results:**

In 9/66 cases, (14 %) proton plans were superior for all dose-volume parameters. Higher T stages benefited more from protons in Profile I, especially tumors located within 3 mm or less from the optic nerve. In Profile II, only 9/66 cases resulted in a better proton plan. In Profile III, better retina volume sparing was always achievable with protons, with a larger gain for T3 tumors. In Profile IV, protons always reduced the risk of toxicity with a median RBE-weighted EQD2 reduction of 15.3 Gy.

**Conclusions:**

This study reports the first side-by-side imaging-based planning comparison between protons and SRT for UM patients. Globally, while protons appear almost always better regarding the risk of optic-neuropathy, retinopathy and dry-eye syndrome, for other toxicity like neovascular glaucoma, a plan comparison is warranted. Choice would depend on the prioritization of risks.

## Highlights

• A side-by-side comparative study between stereotactic radiotherapy and protons for uveal melanomas.
• Four toxicity profiles have been evaluated reflecting major ocular complications.
• Only 14% of cases had superior proton doses for all metrics.
• Protons decrease the risk of optic-neuropathy, retinopathy, and dry-eye syndrome.
• A plan comparison is warranted to address the risk of neovascular glaucoma.

## 1 Introduction

Uveal melanoma (UM) patients are commonly treated with either surgery, photon-based stereotactic radiotherapy (SRT) [1], [2], [3], [4], [5], [6], [7], [8], [9], [10], [11], plaque brachytherapy, or proton therapy. UM is a standard indication for proton therapy [12] with a 5-year local control rate of more than 90 % worldwide [13], [14], [15], [16], [17]. Brachytherapy [18], [19], [20], [21] and SRT [2], [5], [7], [10], [22], [23] result in 5-year local control of 75–95 %. The selection among those techniques is based on the availability of a specific treatment modality, the ocular team experience, patient’s preferences, and/or reimbursements. Differences in outcomes are still debated [24], [25] and up to 70 % of adverse events have been reported after any radiotherapy [26]. As more centers treat UM with photon-based SRT and the number of proton centers worldwide increases, the patient selection based on the potential trade-off in toxicities needs to be addressed. The choice of the optimal technique should account for both the risk of post-treatment complications [26] and quality-of-life [27]. However, direct photon-proton plan comparison studies are not possible due to differences in treatment planning. Proton planning is historically based on a generic geometrical model [28], while SRT utilizes a CT-based planning approach.

This study aimed to evaluate the differences in dose-volume metrics between SRT and proton therapy for UM patients using a CT-based *in-silico* planning comparative study. Four toxicity-specific profiles, representing the most clinically relevant complications, were compared to evaluate the potential benefit of each treatment option.

## 2 Materials and methods

### 2.1 Study population

Clinical baseline characteristics and treatment details of 66 UM patients (40 left, 26 right eyes) treated between 2016 and 2021 at Erasmus Medical Center (Rotterdam, The Netherlands) with SRT using a robotic CyberKnife M6 (Accuray, Sunnyvale, CA, USA) are summarized in Table 1. The local Ethics Committee approved the study (MEC-2021-0454). Tumors were classified using the American Joint Committee on Cancer (AJCC) staging [29]: 15 T1, 25 T2 and 26 T3. The median tumor apical height was 5.5 mm (range: 1.8–12.8 mm) and median volume was 0.4 cm^3^ (range: 0.1–1.7 cm^3^). Tumor apical height and largest basal diameter were retrieved from B-scan ultrasonography before irradiation. A planning CT was acquired at straight gazing angle with a voxel resolution of (0.59x0.59x1) mm^3^. Anterior segment, retina (1-mm inset from the external eye globe until ora serrata), eye globe, vitreous body, optic nerve, optic disc, and lacrimal gland were manually contoured. As the macula was not discernible on CT or MRI, it was geometrically reconstructed based on fundus photography. For patients suffering from vitreous hemorrhage, the macula was defined at the intersection between the optical axis and retina. Registered MRI was used for delineation of the Gross Tumor Volume (GTV) when available.

**Table 1:** Baseline patient characteristics and treatment details.

**Study population**						
Age at diagnosis (mean ± SD, in years)					67.3 ± 13.2	
Gender (n(%))	Female				27 (41 %)	
	Male				39 (59 %)	
Eye (n(%))		OS			40 (60.6 %)	
		OD			26 (39.4 %)	

**Tumor characteristics**						
LBD (median [range], in mm), (on US)					11.42 [4.34; 16.84]	
Apical height (median [range], in mm), (on US)					5.51 [1.84; 12.80]	
Tumor size classification AJCC T (n(%))						
	T1				15 (22.7 %)	
	T2				25 (37.9 %)	
	T3				26 (39.4 %)	
Size of GTV (median [range], in cm^3^)^a^					0.36 [0.07; 1.65]	
*Minimum distance tumor edge to organs-at-risk (median [range], in mm)*						
	Fovea (on US)				2.00 [0.00; 12.00]	
	Optic nerve ^a^				2.59 [0.00; 18.84]	
	Optic disc ^a^				1.79 [0.00; 17.74]	
	Anterior segment ^a^				5.67 [0.00; 13.98]	
	Lacrimal gland ^a^				4.06 [0.00; 20.03]	

**Treatment details**						
*Fractionated stereotactic radiotherapy (SRT)*						
	Prescription dose (RBE-weighted, in Gy)				50	
	No. of fractions				5	
	No. of beams (mean ± SD)				62.86 ± 21.66	
	Iris collimator				59/66 cases (sizes: 7.5, 10, 12.5, 15 and/or 20 mm)	
	Multileaf collimator				7/66 cases	
	Beam-on time ^b^ (mean ± SD, in min)				21.65 ± 3.62	
	Gazing angle				Straight **^c^**	
	Monitor Units (delivered plans, mean ± SD)				2951.83 ± 964.81	
*Proton therapy*						
	Prescription dose (RBE-weighted, in Gy)				60	
	No. of fractions				4	
	No. of beams				1	
	Beam-on time				∼ 1 min	
	Ocular motility limits (gazing angle)				[−30; 30] degrees	
	Monitor Units				NA **^d^**	

**^a^** CT-based measurements; **^b^** Including a 5 min patient setup; **^c^** Fixation light usually placed at the center of the pupil of the treated or healthy eye; **^d^** Cannot be determined from the clinical TPS, within current clinical practice in ocular proton therapy using a dedicated eyeline.

*Abbreviations:* SD = Standard Deviation; LBD = largest basal diameter; US = ultrasound; AJCC = American Joint Committee on Cancer staging consensus [30]. SRT = fractionated stereotactic radiotherapy; GTV = gross tumor volume; TPS = treatment planning system.

### 2.2 Stereotactic radiotherapy

The relative biological effectiveness (RBE)-weighted dose of 50 Gy [23] (5x10 Gy) was prescribed to the 80 % isodose encompassing the Planning Target Volume (PTV) according to the International Commission of Radiation Units and Measurements (ICRU) Report 91 guidelines [30]. PTV included GTV with a 2-mm isotropic margin derived upon a multidisciplinary local consensus based on the assessment of uncertainties. At least 98 % of PTV received 95 % of the prescribed dose, resulting in a PTV near-maximum dose of 62.5 Gy (RBE-weighted = 1.0). Dose was calculated in Accuray Multiplan™ (Accuray, Sunnyvale, CA, USA) and delivered with a robotic CyberKnife M6 using either an iris collimator (range: 7.5 to 20 mm) or a multi-leaf collimator. To compensate for intrafraction patient motion, the 6D skull tracking method was used with tight rotational and translational boundaries. To mitigate eye setup errors, gating using a modified version of the Rotterdam Gill-Thomas-Cosman frame [31] with the camera and LED placed on an arch attached to a double-shell mask was employed.

### 2.3 Simulation of proton therapy

The prescription dose for proton therapy was 60 Gy (4x15 Gy, RBE-weighted = 1.1) within the treatment field where 100 % of dose was defined from the average dose within this treatment field according to ICRU Report 78 [32], resulting in at least 95 % of GTV receiving 90 % of the prescribed dose. Following an international consensus in ocular proton therapy [13], [14], [33], [34], [35], [36], [37], a 2.5-mm expansion was applied proximally and distally along the beam central axis to define the Spread-Out Bragg Peak. Laterally, a collimator encompassing the GTV with 2.5-mm margin was used, enabling at least 50 % of dose within the aperture. An optimal gazing angle to minimize organs-at-risk exposure and maximize treatment conformity was selected. Since the CTs were not performed at the optimal gazing angle, a composite transformation overlaid the simulated proton dose onto the planning CTs. The detailed description of the in-house dose algorithm used in this study is reported elsewhere [38], [39].

### 2.4 Plan comparison and statistical analysis

Analysis accounted for the AJCC T staging, toxicity-specific profiles, and distance to the optic nerve. Four post-treatment complications profiles and relevant dose-volume parameters were evaluated [16], [40], [41], [42]:

• **Profile I**) Maculopathy, optic-neuropathy, and visual acuity impairment: D_2%_, D_mean_ and V_30Gy_ to the optic nerve, D_mean_ to the optic disc, and D_2%_ to the macula;
• **Profile II**) Neovascular glaucoma: D_2%_ and D_mean_ received by the anterior segment, and the D_2%_ to the optic nerve;
• **Profile III)** Radiation-induced retinopathy: D_20%_ dose and V_5Gy_, V_10Gy_, V_20Gy_, V_30Gy_ volumes to the retina;
• **Profile IV)** Dry-eye syndrome: D_2%_ and D_mean_ to the lacrimal gland.

For Profile I, all patients were categorized based on the distance from the tumor edge to the optic nerve, using a 3-mm threshold corresponding to a standard value globally reported for lateral and distal penumbrae with ocular beamlines [43], [44], [45]. The complications severity ranking followed our institutional multidisciplinary consensus.

A radiobiological equivalent dose of 2-Gy fractions (EQD2) with a 10 Gy α/β ratio for UM was calculated: RBE-weighted EQD2 prescribed dose was 83 Gy for SRT and 125 Gy for protons. D_2%_, D_20%_ and D_mean_ were also converted to RBE-weighted EQD2 using α/β of 2 Gy for the optic nerve, optic disc, macula, retina, anterior segment, and 3 Gy for the lacrimal gland. These values were based on the values reported by Joiner *et al* [46] and determined through a multidisciplinary consensus at HollandPTC (Delft, The Netherlands) to be used clinically. Dose-volume calculations for SRT and protons were performed with the in-house software Mattherhorn to avoid bias. Differences in RBE-weighted EQD2 dose between SRT and protons were calculated for every patient of the present cohort; and the EQD2-median of those differences used for comparison. This study followed the RATING guidelines [47].

3D retina dose surface maps, incorporating real retinal CT-based imaging, were created to enable a quantitative anatomical comparison in terms of exposed area. Additionally, 2D planar maps for surface dose were generated following the method described by Hoogeman *et al* [48]. Mercator dose projections of the retina surface were made from the centroid of each plane in the vitreous body with the outer retina perimeter of each plane segmented using a fixed number of 360 points evenly spaced. The 2D maps were expressed in RBE-weighted dose and RBE-weighted EQD2 dose.

Two-sided Wilcoxon signed-rank tests were used to evaluate the statistical significance of observed differences. Because of multiple tests, the significance was defined as p < 0.01. Analyses were performed using Python v3.7 and the SciPy package.

## 3 Results

All plans for both modalities achieved adequate target coverage. For all patients, 98 % of PTV received at least 95 % of the SRT dose. With protons, GTV received at least 90 % of the prescribed dose. Median RBE-weighted GTV D_mean_ was 59.6 Gy (range: 57.0–61.3 Gy) for SRT and 59.0 Gy (range: 57.6–59.6 Gy) for protons (Table 1). Average RATING score calculated by two authors (P.T, E.F.) was 82 %.

**Fig. 1:**
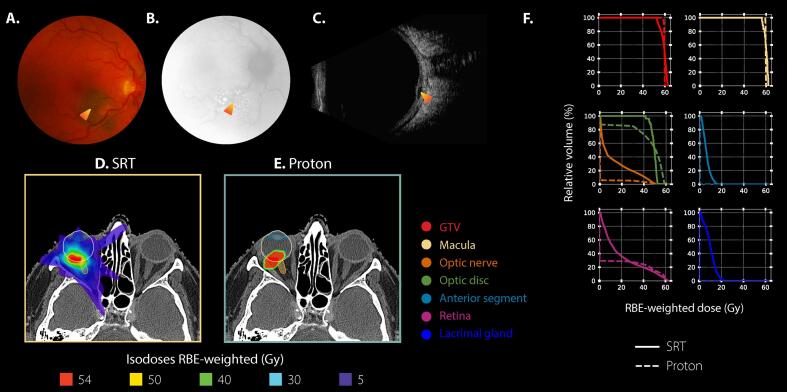
**Case example** of an 80-year-old female patient diagnosed with an AJCC T1-staged choroidal melanoma in her right eye. **A, B, C)** Ophthalmic images at diagnosis. The orange-shaded arrow indicates the lesion. **A:** Fundus photography. **B:** Fluorescent angiography. **C:** B-scan ultrasound. Dimensions on ultrasound were of 7.2 mm for largest basal diameter and of 2.1 mm for tumor apical height. **D, E)** Axial dose distributions shown on the planning CT in respect to the gazing angle hold by the patient during the scan. For proton simulation, a gazing angle [Ψ = 30 degrees; ɸ=30 degrees] was considered. Ψ represented any elevation/depression of the eye, whereas ɸ represented any ab-/adduction. **F)** DVH results for both modalities, stereotactic radiotherapy (SRT) in solid line and proton in dashed line. Results are presented for a total treatment dose, in RBE-weighted dose (in Gy), or percentage of irradiated volume (%-point). (For interpretation of the references to colour in this figure legend, the reader is referred to the web version of this article.)

**Fig. 2:**
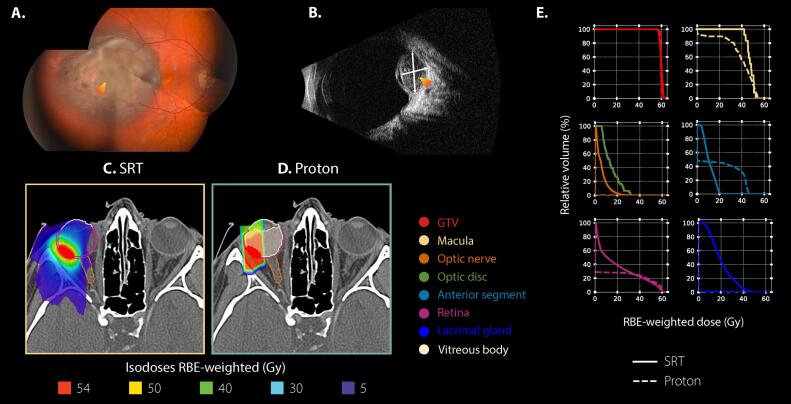
**Case example** of a 21-year-old female patient diagnosed with an AJCC T2-staged choroidal melanoma in her right eye. **A, B)** Ophthalmic images at diagnosis. The orange-shaded arrow indicates the lesion. **A:** Fundus photography. **B:** B-scan ultrasound. Dimensions on ultrasound were of 8.4 mm for largest basal diameter and of 5.3 mm for tumor apical height. **C, D)** Axial dose distributions shown on the planning CT in respect to the gazing angle hold by the patient during the scan. For proton simulation, a straight gazing angle [Ψ = 0 degrees; ɸ=0 degrees] was considered. Ψ represented any elevation/depression of the eye, whereas ɸ represented any ab-/adduction. **E)** DVH results for both modalities, stereotactic radiotherapy (SRT) in solid line and proton in dashed line. Results are presented for a total treatment dose, in RBE-weighted dose (in Gy), or percentage of irradiated volume (%-point). (For interpretation of the references to colour in this figure legend, the reader is referred to the web version of this article.)

**Fig. 3:**
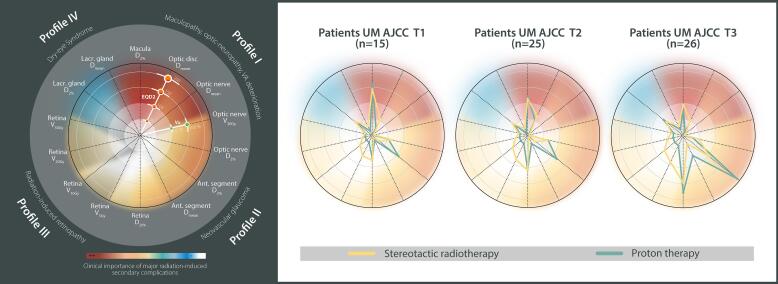
**AJCC-based dosimetric spider maps. Left panel)** Graphical representation of dose-volume metrics for every parameter included into each toxicity-specific Profile I to IV. Concentric rings are scaled in RBE-weighted EQD2 dose (in Gy), or percentage of irradiated volume (Vx, %-point). The severity ranking of complications followed institutional multidisciplinary consensus for stereotactic radiotherapy and proton therapy. **Right panels)** Population-averaged spider maps for T1, T2 and T3 uveal melanoma (UM) patients of the cohort. Dose-volume metric values are expressed for a total treatment dose and displayed for fractionated stereotactic radiotherapy plans (in yellow) and proton plans (in green). (For interpretation of the references to colour in this figure legend, the reader is referred to the web version of this article.)

Patient examples and the associated comparisons are presented in Fig. 1 and Fig. 2. Comparative evaluation of dose-volume parameters is reported in Table 2 and Figure S1 in Supplementary Material, and graphically shown in Fig. 3 using AJCC-specific population-averaged spider maps. Superior proton plans for all 14 dose-volume metrics were in 9/66 patients. In 57/66 patients, some dose-volume parameters were favorable to SRT and some to protons, depending on the evaluated dose-volume metric.

**Table 2:** Dosimetric findings (RBE-weighted dose or RBE-weighted EQD2 dose, or anatomical irradiated volume) across the cohort of patients. All values are expressed for a total treatment dose.

			**Median RBE-weighted dose [range]**				**RBE-weighted EQD2-median dose [range]**				**RBE-weighted EQD2-median difference [IQR]**		**RBE-weighted EQD2-Comparison**
			SRT		Protons		SRT		Protons		Δ (Protons − SRT)*		Wilcoxon test for paired samples
GTV	D_mean_	[Gy]	**59.6**	[57.0; 61.3]	**59.0**	[57.6; 59.6]	**109.0**	[101.6; 113.6]	**121.7**	[116.9; 123.7]	**12.9**	[10.9; 14.1]	*p =* ns
	V_54Gy_	[%]	**100.0**	[100.0; 100.0]	**99.9**	[98.5; 100.0]	**100.0**	[100.0; 100.0]	**99.9**	[98.5; 100.0]	**−0.1**	[−0.3; 0.0]	***p* < 0.01**

Macula	D_2%_	[Gy]	**44.1**	[5.7; 62.0]	**42.3**	[0.0; 61.1]	**119.1**	[4.5; 222.9]	**133.2**	[0.0; 263.8]	**−5.5**	[−25.8; 45.3]	***p* = 0.04**

Optic nerve	D_2%_	[Gy]	**20.6**	[1.3; 56.2]	**0.0**	[0.0; 61.1]	**31.5**	[0.7; 186.1]	**0.0**	[0.0; 264.0]	**−18.0**	[−31.8; −4.6]	***p* < 0.01**
	D_mean_	[Gy]	**6.4**	[0.5; 21.6]	**0.0**	[0.0; 21.6]	**5.2**	[0.3; 34.2]	**0.0**	[0.0; 40.0]	**−4.6**	[−7.5; −2.7]	***p* < 0.01**
	V_30Gy_	[%]	**0.0**	[0.0; 34.1]	**0.0**	[0.0; 37.0]	**0.1**	[0.0; 34.1]	**0.0**	[0.0; 37.0]	**0**	[−2.9; 0.0]	***p* < 0.01**

Optic disc	D_mean_	[Gy]	**20.5**	[1.4; 59.5]	**0.7**	[0.0; 59.6]	**31.3**	[0.8; 206.9]	**0.4**	[0.0; 251.6]	**−21.4**	[−45.7; −12.9]	***p* < 0.01**

Anterior segment	D_2%_	[Gy]	**17.3**	[5.1; 60.8]	**46.7**	[0.0; 59.7]	**23.6**	[3.9; 215.6]	**157.3**	[0.0; 252.3]	**114.5**	[48.4; 140.6]	***p* < 0.01**
	D_mean_	[Gy]	**8.1**	[1.3; 41.3]	**14.5**	[0.0; 49.1]	**7.3**	[0.7; 105.7]	**20.4**	[0.0; 175.5]	**9.0**	[−4.8; 43.0]	***p* < 0.01**

Retina	D_20%_	[Gy]	**43.5**	[17.5; 56.4]	**41.5**	[0.0; 57.6]	**116.3**	[24.1; 187.2]	**125.8**	[0.0; 236.1]	**16.0**	[−27.5; 51.0]	*p* = ns
	V_5Gy_	[%]	**87.3**	[55.1; 100.0]	**28.6**	[8.3; 53.6]	**87.3**	[55.1; 100.0]	**28.6**	[8.3; 53.6]	**−53.7**	[−62.5; −46.3]	***p* < 0.01**
	V_10Gy_	[%]	**60.9**	[34.0; 100.0]	**28.2**	[8.1; 51.6]	**60.9**	[34.0; 100.0]	**28.2**	[8.1; 51.6]	**−32.7**	[−39.1; −26.4]	***p* < 0.01**
	V_20Gy_	[%]	**39.3**	[17.1; 85.7]	**27.7**	[7.8; 50.3]	**39.3**	[17.1; 85.7]	**27.7**	[7.8; 50.3]	**−13.5**	[−16.7; −9.5]	***p* < 0.01**
	V_30Gy_	[%]	**30.1**	[11.2; 54.3]	**25.5**	[7.3; 48.6]	**30.1**	[11.2; 54.3]	**25.5**	[7.3; 48.6]	**−4.9**	[−8.0; −1.9]	***p* < 0.01**

Lacrimal gland	D_2%_	[Gy]	**29.2**	[1.9; 58.1]	**0.0**	[0.0; 61.0]	**51.6**	[1.3; 169.8]	**0.1**	[0.1; 222.6]	**−9.1**	[−32.2; 21.1]	*p* = ns
	D_mean_	[Gy]	**16.7**	[0.6; 45.9]	**0.0**	[0.0; 56.6]	**21.2**	[0.4; 111.6]	**0.0**	[0.0; 194.1]	**−15.3**	[−27.7; −4.0]	***p* < 0.01**

* In respect to the organs-at-risk, negative values are favorable to proton therapy.

Abbreviations: SRT = fractionated stereotactic radiotherapy; GTV = gross tumor volume; ns = non-significant; IQR = Interquartile range.

For Profile I, EQD2-median differences between both modalities were all statistically significant in favor of protons, except for the maculopathy (Table 2). In 36/66 patients, the tumor border was located within 3-mm distance from the optic nerve, with a median minimum distance of 1.2 mm. All dose differences were favorable to protons: EQD2-median reduction of 44.1, 6.1 and 30.5 Gy for optic disc D_mean_, optic nerve D_mean_ and optic nerve D_2%_, respectively (Tables S2). The higher AJCC T staging, the larger the dosimetric gain, with a maximum EQD2-median D_mean_ to the optic disc of 41.2 Gy for T1 and 72.0 Gy for T3 tumors. For the other 30/66 patients, the median minimum distance to the tumor edge was 5.6 mm and the proton EQD2-median optic nerve D_2%_ reduction was 17.8 Gy (Tables S2). Regarding AJCC T staging, EQD2-median macula D_2%_ was reduced with protons by 7.0 Gy for T1 tumors, 11.1 Gy for T2 tumors, and 19.9 Gy for T3 tumors. Independent of tumor location, there was no median optic nerve V_30Gy_ difference observed between protons and SRT.

For Profile II, an EQD2-median anterior segment D_2%_ and D_mean_ reduction of 114.5 Gy (p < 0.01) and 9.0 Gy (p < 0.013), respectively, was achievable with SRT over all patients (Table 2). Protons decreased the optic nerve D_2%_ by an EQD2-median of 18.0 Gy (p < 0.01) compared to SRT. At patient’s level, 9/66 cases had better dose distribution with protons based on the optic nerve D_2%_, and the anterior segment D_2%_ and D_mean_. A minimum tumor edge to optic nerve distance was > 3-mm in 8/9 cases with variability in tumor patterns.

**Fig. 4:**
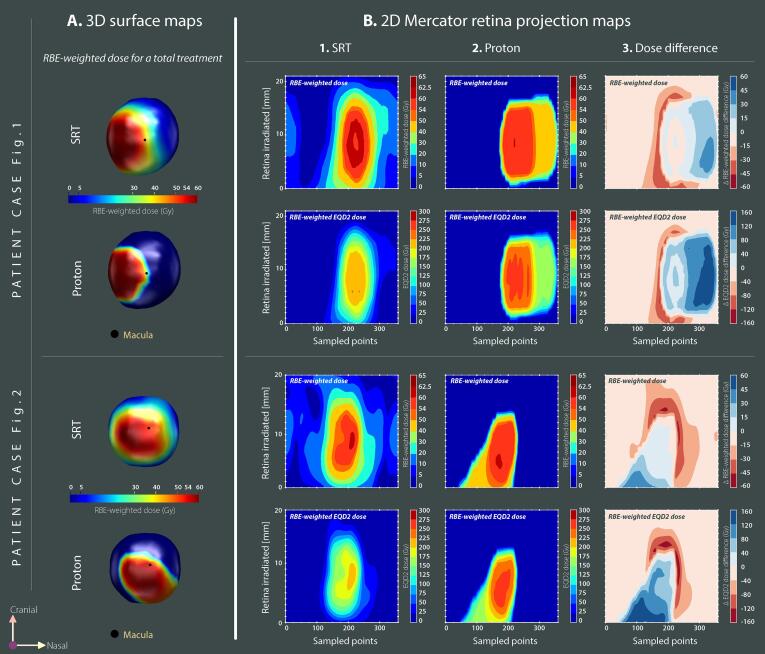
**Retina maps** for patients referenced in Fig. 1 and Fig. 2, respectively. **A)** 3D dose surface maps displayed for a total treatment dose for both modalities (RBE-weighted, in Gy). Camera view is located at the back of the eye. **B)** Planar equivalence of the 3D maps. Mercator projection maps are expressed in RBE-weighted dose (upper panels) and when using an equieffective RBE-weighted dose of 2 Gy fractions, EQD2 (lower panels). Y-axis represents the axial slices from caudal to cranial. Numbering of the sampled dose points is performed along the X-axis, a fixed value of 360 equiangular points in each plane of the vitreous body was used. **B.1)** Fractionated stereotactic radiotherapy plan (SRT). **B.2)** Proton plan. **B.3)** Difference between SRT and protons. Negative values (red zones) are in favor of proton therapy. (For interpretation of the references to colour in this figure legend, the reader is referred to the web version of this article.)

For Profile III, large variations were observed across all patients, with retina D_20%_ sometimes favorable to SRT and sometimes to protons, but not statistically significant due to interpatient variability (Figure S2). Importantly, retina D_20%_ was linked to AJCC staging, suggesting an advantage with protons for small T1 tumors with an EQD2-median dose reduction of 14.2 Gy, but a median increase of 42.0 Gy for T3 tumors. There was a tendency of a more pronounced retina volume sparing for T3 tumors compared to T1-T2 tumors with protons. Since dose-volume-histograms lose geographical information, Mercator projections enabled visualizing the differences between modalities at a patient’s basis. Fig. 4 shows local EQD2 differences between blue zones, in favor of SRT, and red zones, in favor of protons. For the two patients represented, the blue zone appears similar to the red zone, despite lower retina V_5Gy_ to V_20Gy_ with protons (Fig. 1, Fig. 2, Fig. 3).

For Profile IV, an EQD2-median lacrimal gland D_mean_ reduction of 15.3 Gy was observed with protons (p < 0.01) for all patients. AJCC staging had no impact. As expected, larger dose differences were observed for the 42 temporal tumors (Table S3 & Figure S4). For this subgroup, the proton D_mean_ EQD2-median reduction was 22.8 Gy. At individual level, 36/42 patients presented a dosimetric gain with protons. For the 24 nasal tumors, the proton D_mean_ EQD2-median reduction was 5.5 Gy. At individual level, all 24 patients presented a dosimetric gain with protons. Interestingly, lacrimal gland D_2%_ showed large interpatient variability with no significant differences at Wilcoxon test (Figure S3).

## 4 Discussion

This study evaluated the differences in dose-volume metrics between protons and CyberKnife-based SRT for the same cohort of UM patients. Such comparison is *a priori* difficult because of differences in imaging for planning (ultrasound for protons vs. CT for SRT) [28], [49], [50], [51] and in underlying technologies. Moreover, treatment fractionation, dose prescription and RBE also differ, making direct comparison challenging. Therefore, an isoeffective CT-based planning process with both specific GTVs receiving an RBE-weighted D_mean_ of 60 Gy was implemented to ensure an objective comparison. In general, GTV coverage with both protons and SRT was 100 %, except for a few proton cases where coverage was marginally less than 100 %, attributed to uncertainties in calculating the minimum dose due to the finite voxel size. The RBE-weighted EQD2-median dose were much higher for protons. Further discussions on prescription protocols for imaging-based planning in ocular proton therapy are needed to address these differences.

A correlation between radiation-related complications, the size of the tumor, and its proximity to sensitive ocular structures like the optic nerve or the macula/fovea was previously reported [52]. The vision (Profile I) might be irreversibly compromised after radiation-induced maculopathy and optic-neuropathy [53]. In our data, proton plans were dosimetrically superior to SRT in parameters related to vision impairment [50] with a benefit more pronounced for T3 tumors than T1-2 and less pronounced for tumors located adjacent to the optic nerve/disc, similar to other studies [17], [54]. In a retrospective, non-randomized study comparing radiosurgery with protons, Sikuade *et al* reported on better visual acuity preservation with proton compared to radiosurgery [55]. Interestingly, while it is well-established that doses above 50 Gy lead to optic neuropathy [53], the optic nerve continues to be delineated as a straight tube of a few millimeters in diameter for proton planning. This segment of the optic nerve is particularly vulnerable to radiation damage, owing to its lack of myelin sheathing [56]. In our study, imaging-based planning was used for both treatment modalities, more precise evaluation of the dose distribution along the entire length of the optic nerve was possible. Such an accuracy in dose assessment during treatment planning could potentially contribute to the preservation of some visual function [26], [57]. Additionally, biomechanical modelling could improve accuracy of gazing-angle specific optical nerve shape and position. A physical wedge and/or combined with the use of a bolus can also reduce dose in specific cases [56], [58].

Neovascular glaucoma may lead to enucleation after radiotherapy, generally triggered following local recurrence, tumor necrosis followed by toxic tumor syndrome, and/or painful neovascular glaucoma [37], [59], [60], all caused by radiation damages to the anterior chamber, but also to the posterior chamber [16], [59], [61], [62]. Overall, RBE-weighted EQD2-median D_2%_ and D_mean_ values to the anterior segment were always better in SRT. Looking at both segments, protons demonstrated dosimetric advantage in 9/66 patients, with 8 patients having tumors located further than 3-mm from the optic nerve, similar to literature [60]. Both distances to organs-at-risk and dose-volume parameters should be considered for the best treatment option selection regarding the risk of neovascular glaucoma. Additionally, employing multiple beams with protons instead of a single one, as recently suggested [39], might be a promising approach for improved sparing of the anterior region.

For radiation-induced retinopathy (Profile III), our data revealed better retina volume sparing with protons, especially for higher tumor stages (Fig. 3). Retinopathy has high incidence rates after any radiotherapy [22], [63], depending on the total dose, fractionation, tumor diameter, and retina dose [40], [64], [65], [66]. The tumor diameter may be an indirect indicator of the amount of retina surface irradiated. Mercator dose projection maps were used to better understand the geographical location of the dose. The concept is similar to the retinal diagram originally proposed for episcleral brachytherapy where a rasterized polar retina map is displayed along with the isodoses [67] and in conjunction with fundus image it can better identify the retina area at risk of damage [68]. The retinal diagram represents a 2D surface mapping of a sphere, applicable for geometrical model-based ocular treatment. Retina contoured on CT images is, however, irregular. Unfolding an irregularly shaped 3D retina into a 2D surface map is not a trivial task. Mercator maps enable patient-specific evaluation and therefore may be more accurate for dose-toxicity relationship studies for radiation-induced retinopathy than retinal diagrams.

Eye dryness (Profile IV) is mainly due to radiation damage of the lacrimal gland. For protons, due to the sharpness of the penumbrae, reducing the lacrimal gland dose was achievable compared to the SRT doses. It is note-worthy that additional research could enhance lacrimal gland and eyelid dose sparing during proton therapy. For instance, the head tilt might be incorporated into the simulation process and be beneficial in some selected cases.

The study has several limitations. Firstly, as UM is a rare disease, a small number of patients was available, limiting generalization of conclusion. Similar comparisons with even smaller number of patients were performed previously by Höcht *et al* [50] (ten patients) and Weber *et al* [49] (one simulated patient). Secondly, the patient selection (single-institute SRT) may introduce bias. Inclusion of other treatment modalities, such as brachytherapy, may alter the conclusions, especially for small tumors where brachytherapy is generally the treatment-of-choice. Thirdly, the selection of constraints was based on literature [16], [40], [41], [42] due to the lack of solid knowledge on dose-volume effects for ocular radiotherapy. Many established dose-volume tolerance levels included in various guidelines, e.g. QUANTEC [53], [69] or RTOG [70], [71], [72], cannot be applied for hypo-fractionated regimen. Only one normal tissue complication probability model for choroidal melanoma post-proton therapy exits [42]. Additionally, there are significant uncertainties regarding the α/β-ratios for various ocular structures and their radiation-induced effects. A broad spectrum of α/β-values is reported [73], varying from 2.6 and 12.1 Gy across different UM cell lines, thus potentially leading to significant differences in the prescribed RBE-weighted EQDx doses in current clinical radiotherapy. It is important to further explore tissue response data for hypo-fractionated treatments, specifically UM cell lines, concerning different high-dose fractionation schemes with SRT and proton therapy. The investigation of radiation dose achieving the best treatment outcomes in respect to local tumor control or ocular morbidity was beyond the scope of this study. Lastly, the findings of this research were specifically based on the beam properties of the HollandPTC eyeline [44]. It is important to emphasize that variations in beam quality across eyelines, especially in terms of lateral and distal penumbrae, might impact the conclusions of this research [58], [74]. Unlike proton eyelines, the beam properties of CyberKnife systems are consistent across different centers [75].

In conclusion, this study represents the first imaging-based comparison for real-life UM patients between SRT and protons. Looking at the population as a whole, protons offer dosimetric advantages over fractionated stereotactic treatments, however there may be individual patients for whom this is not the case. This research supports the necessity for a plan comparison strategy to address differences in toxicities and help ocular oncology teams decide the appropriate treatment choice together with the patient when both options are available.

## Funding statement

This research was co-funded by the research program PROTONS4Vision (Grant NWO 14654), which was financed by the Netherlands Organization for Scientific Research (NWO), Technology Foundation STW, the Top consortium for Knowledge & Innovation (TKI-HTSM), and Varian, a Siemens Healthineers Company, Palo Alto, California, USA.

## CRediT authorship contribution statement

**Emmanuelle Fleury:** Conceptualization, Methodology, Formal analysis, Writing – original draft. **Jean-Philippe Pignol:** Methodology, Writing – review & editing, Supervision. **Emine Kiliç:** Conceptualization, Validation, Resources, Visualization. **Maaike Milder:** Conceptualization, Validation, Resources, Visualization. **Caroline van Rij:** Conceptualization, Validation, Resources, Visualization. **Nicole Naus:** Conceptualization, Validation, Resources, Visualization. **Serdar Yavuzyigitoglu:** Conceptualization, Validation, Resources, Visualization. **Wilhelm den Toom:** Conceptualization, Validation, Resources, Visualization. **Andras Zolnay:** Conceptualization, Validation, Resources, Visualization. **Kees Spruijt:** Conceptualization, Validation, Resources, Visualization. **Marco van Vulpen:** Conceptualization, Validation, Resources, Visualization. **Petra Trnková:** Writing – review & editing, Supervision. **Mischa Hoogeman:** Project administration, Funding acquisition, Conceptualization, Methodology, Writing – review & editing, Supervision.

## Declaration of Competing Interest

The authors declare the following financial interests/personal relationships which may be considered as potential competing interests: **E. Fleury:** The Department of Radiotherapy (Erasmus Medical Center Cancer Institute, The Netherlands) has research collaborations with Elekta AB, Stockholm, Sweden, Accuray Inc., Sunnyvale, CA, USA, Varian, Palo Alto, CA, USA, RaySearch Laboratories, Stockholm, Sweden, outside the submitted work. **Prof. dr. Pignol:** Prof. dr. Pignol was senior vice president, chief medical and technology officer at Accuray Inc., Sunnyvale, CA, USA until February 2023. **Prof. dr. Hoogeman:** Prof. dr. Hoogeman reports grants from Netherlands Organization for Scientific Research, grants from Varian, a Siemens Healthineers Company, Palo Alto, California, USA, during the conduct of the study; being a Member of advisory board Accuray, Sunnyvale, USA; being a participant/presenter at Accuray Thinktank Meeting on Prostate cancer, outside the submitted work; and The Department of Radiotherapy (Erasmus Medical Center Cancer Institute, The Netherlands) has research collaborations with Elekta AB, Stockholm, Sweden, Accuray Inc., Sunnyvale, CA, USA, Varian, Palo Alto, CA, USA, RaySearch Laboratories, Stockholm, Sweden, outside the submitted work.
