# Supplementary material for: Comparison of stereotactic radiotherapy and protons for uveal melanoma patients
Source: Phys Imaging Radiat Oncol. 2024 Jun 26;31:100605. doi: 10.1016/j.phro.2024.100605 (PMC11268348; doi:10.1016/j.phro.2024.100605)
Supplement: Supplementary Data 1 [file mmc1.pdf]

Comparison of Stereotactic Radiotherapy and Protons for Uveal Melanoma Patients

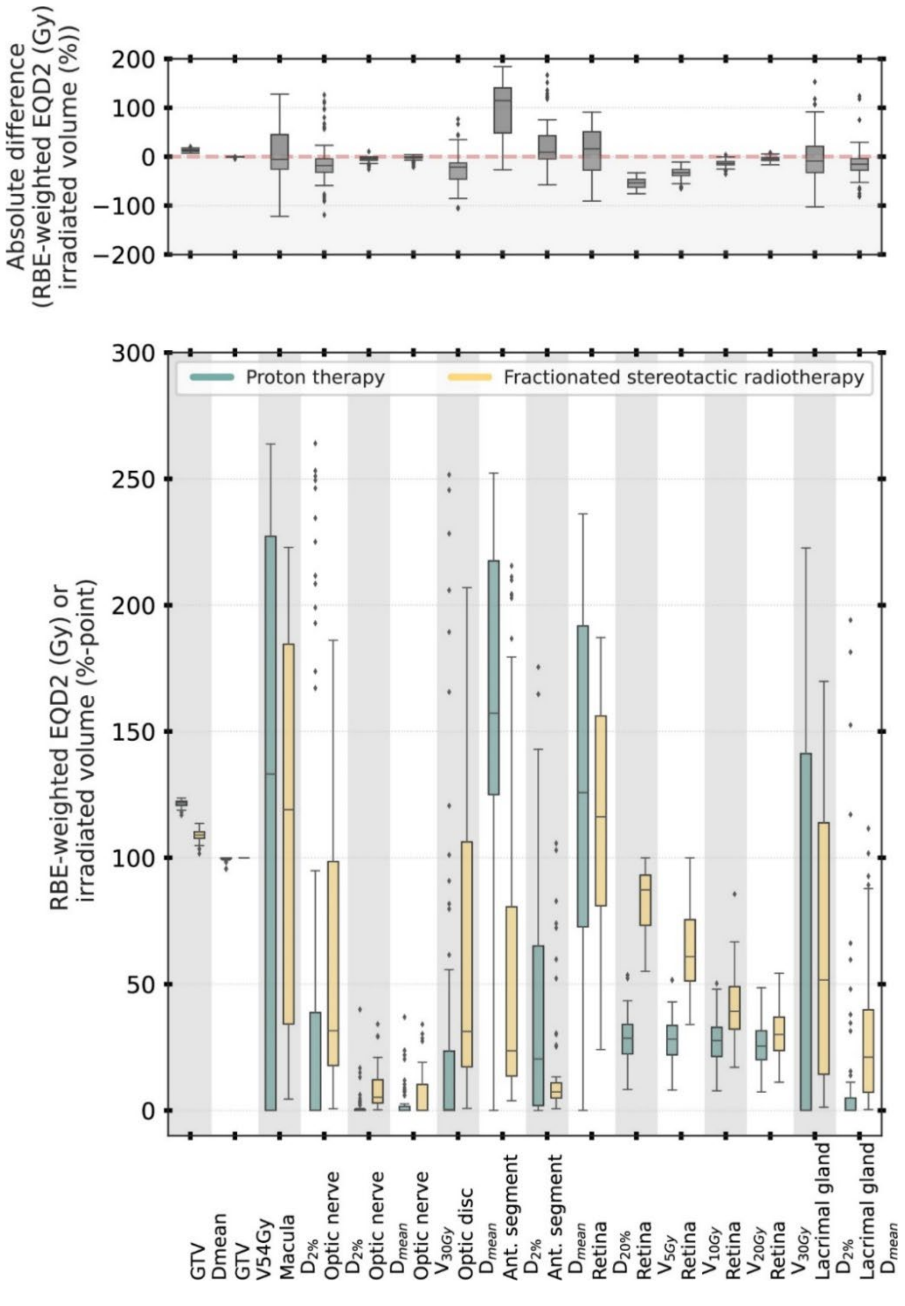

Figure S1: *(Top row)* Boxplots of the observed absolute differences (RBE-weighted EQD2 dose in Gy or %-point of irradiated volume) between stereotactic radiotherapy and protons. Negative values indicate a dosimetric advantage for protons over stereotactic radiotherapy. *(Bottom row):* Boxplots depicting distributions of dosimetric parameter values (RBE-weighted EQD2 dose in Gy or %-point), evaluated for protons (in green) and fractionated stereotactic radiotherapy (in yellow). All values are expressed for a total treatment dose.

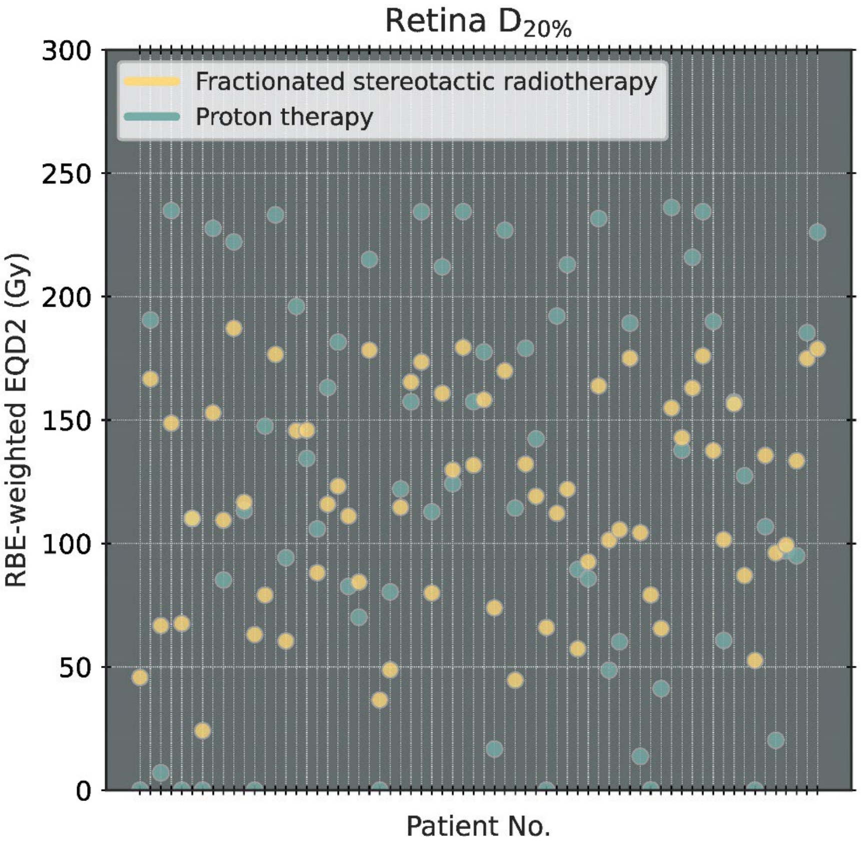

<u>Figure S2:</u> **Dose analysis of the $D_{20\%}$ retina at patient basis.** Each dot represents the RBE-weighted EQD2 dose received by the $D_{2\%}$ of the lacrimal gland for stereotactic radiotherapy (in yellow) and proton therapy (in green).

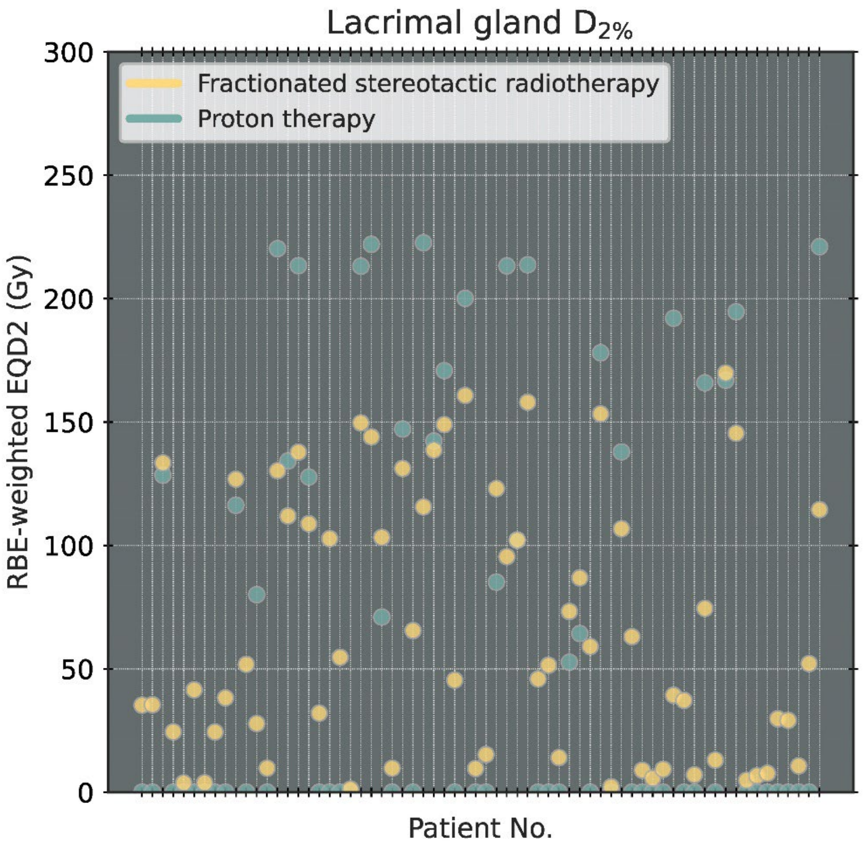

<u>**Figure S3:**</u> **Dose analysis of the $D_{2\%}$ lacrimal gland at patient's basis.** Each dot represents the RBE-weighted EQD2 dose received by the $D_{2\%}$ of the lacrimal gland for stereotactic radiotherapy (in yellow) and proton therapy (in green).

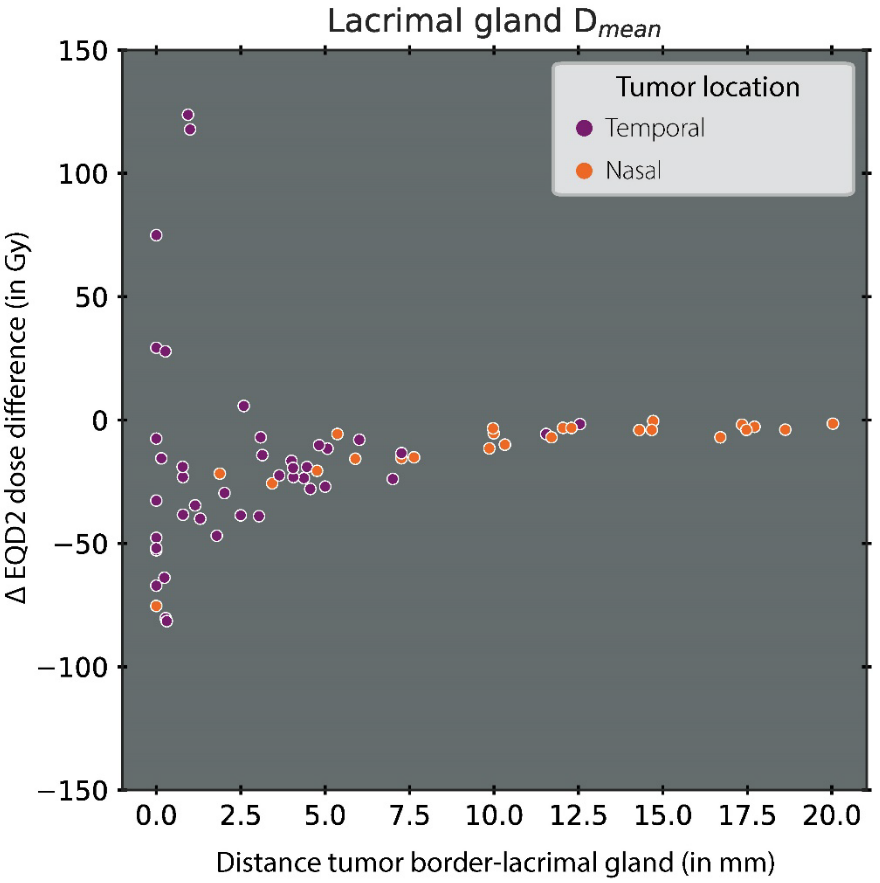

Figure S4: **Dose analysis of the D_{mean} lacrimal gland at patient's basis.** Each dot represents the RBE-weighted EQD2 dose difference received by the D_{mean} of the lacrimal gland between stereotactic radiotherapy and proton therapy in relation to tumor location (nasal tumors (in orange) vs. temporal tumors (in purple)).
