# Supplementary material for: Comparison of stereotactic radiotherapy and protons for uveal melanoma patients
Source: Phys Imaging Radiat Oncol. 2024 Jun 26;31:100605. doi: 10.1016/j.phro.2024.100605 (PMC11268348; doi:10.1016/j.phro.2024.100605)
Supplement: Supplementary Data 2 [file mmc2.pdf]

Comparison of Stereotactic Radiotherapy and Protons for Uveal Melanoma Patients

Tables S1: Toxicity-specific profile I: Maculopathy, optic-neuropathy, visual acuity deterioration.

Subgroup A: Tumors located within 3 mm from the optic nerve.

Subgroup A (n=36)			RBE-weighted EQD2-median dose difference or irradiated volume in % [IQR]	
Macula	$D_{2\%}$		**27.3**	[-25.1; 50.7]
Optic nerve	$D_{2\%}$		**-30.5**	[-50.2; 59.0]
	D_{mean}		**-6.1**	[-12.0; -3.2]
	V_{30Gy}	(in %)	**-2.1**	[-6.7; -0.2]
Optic disc	D_{mean}		**-44.1**	[-70.4; -25.7]

Subgroup A (n=36)				RBE-weighted EQD2-median dose difference or irradiated volume in % [IQR]	
Macula	$D_{2\%}$		T1 *(n=9)*	**36.7**	[14.9; 56.6]
			T2 *(n=16)*	**33.1**	[-20.6; 57.8]
			T3 *(n=11)*	**-15.9**	[-70.9; 35.3]
Optic nerve	$D_{2\%}$		T1	**-31.4**	[-38.9; 4.0]
			T2	**-12.0**	[-34.1; 67.2]
			T3	**-52.0**	[-87.5; 32.4]
	D_{mean}		T1	**-6.2**	[-14.6; -4.3]
			T2	**-3.1**	[-6.3; -2.5]
			T3	**-12.0**	[-14.6; -9.2]
	V_{30Gy}	(in %)	T1	**-0.9**	[-13.1; -0.3]
			T2	**-0.4**	[-3.2; 0.0]
			T3	**-4.0**	[-11.2; -2.9]
Optic disc	D_{mean}		T1	**-41.2**	[-62.2; -36.1]
			T2	**-34.4**	[-46.0; 36.8]
			T3	**-72.0**	[-84.3; -49.7]

***Subgroup B:* Tumors located further away than 3 mm from the optic nerve.**

Subgroup B (n=30)			RBE-weighted EQD2-median dose difference or irradiated volume in % [IQR]	
Macula	$D_{2\%}$		**-10.4**	[-25.4; 12.6]
Optic nerve	$D_{2\%}$		**-17.8**	[-21.0; -12.7]
	D_{mean}		**-4.1**	[-5.8; -2.5]
	V_{30Gy}	(in %)	**0.0**	[0.0; 0.0]
Optic disc	D_{mean}		**-17.4**	[-20.4; -12.5]

Subgroup B (n=30)				RBE-weighted EQD2-median dose difference or irradiated volume in % [IQR]	
Macula	$D_{2\%}$		T1 *(n=6)*	**-7.0**	[-9.3; 18.7]
			T2 *(n=9)*	**-11.1**	[-13.9; -2.4]
			T3 *(n=15)*	**-19.9**	[-30.2; 6.6]
Optic nerve	$D_{2\%}$		T1	**-17.8**	[-20.0; -7.4]
			T2	**-17.0**	[-19.8; -12.6]
			T3	**-19.2**	[-21.3; -14.8]
	D_{mean}		T1	**-2.6**	[-3.8; -1.6]
			T2	**-3.8**	[-4.6; -2.4]
			T3	**-4.9**	[-6.4; -3.7]
	V_{30Gy}	(in %)	T1	**0.0**	[0.0; 0.0]
			T2	**0.0**	[0.0; 0.0]
			T3	**0.0**	[0.0; 0.0]
Optic disc	D_{mean}		T1	**-14.5**	[-19.2; -6.0]
			T2	**-14.6**	[-17.7; -9.7]
			T3	**-18.6**	[-22.3; -14.6]

Tables S2: **Toxicity-specific profile III: Radiation-induced retinopathy.**

All patients (n=66)				RBE-weighted EQD2-median dose difference or irradiated volume in % [IQR]	
Retina	$D_{20\%}$		T1 *(n=15)*	**-14.2**	[-30.4; 14.6]
			T2 *(n=25)*	**-6.8**	[-52.5; 40.4]
			T3 *(n=26)*	**42.0**	[11.4; 58]
	V_{5Gy}	(in %)	T1	**-46.7**	[-50.8; -41.0]
			T2	**-56.0**	[-61.6; -48.1]
			T3	**-58.2**	[-65.4; -47.2]
	V_{10Gy}	(in %)	T1	**-25.6**	[-29.0; -21.9]
			T2	**-32.7**	[-37.3; -26.1]
			T3	**-37.1**	[-47.9;-32.5]
	V_{20Gy}	(in %)	T1	**-9.4**	[-10.3; -6.4]
			T2	**-12.4**	[-15.4; -9.9]
			T3	**-16.3**	[-21.4; -13.2]
	V_{30Gy}	(in %)	T1	**-3.3**	[-4.2; -0.7]
			T2	**-4.5**	[-7.9; -1.8]
			T3	**-6.0**	[-10.1; -4.6]

Table S3: **Toxicity-specific profile IV: Dry-Eye Syndrome.**

All patients (n=66)			RBE-weighted EQD2-median dose difference [IQR]	
Lacrimal gland	$D_{2\%}$	Nasal *(n=24)*	**-9.4**	[-16.5; -5.4]
		Temporal *(n=42)*	**-4.4**	[-38.1; 46.8]
	D_{mean}	Nasal	**-5.5**	[-15.2; -3.3]
		Temporal	**-22.8**	[-38.6; -8.5]
